# Morphology of partial-thickness macular defects: presumed roles of Müller cells and tissue layer interfaces of low mechanical stability

**DOI:** 10.1186/s40942-020-00232-1

**Published:** 2020-07-06

**Authors:** Andreas Bringmann, Jan Darius Unterlauft, Renate Wiedemann, Matus Rehak, Peter Wiedemann

**Affiliations:** Department of Ophthalmology and Eye Hospital, University of Leipzig, Liebigstrasse 10-14, 04103 Leipzig, Germany

**Keywords:** Macular defect, Lamellar hole, Vitreofoveal traction, Epiretinal membrane, Fovea, Müller glia

## Abstract

**Background:**

The pathogenesis of partial-thickness macular defects and the role of Müller glial cells in the development of such defects are not well understood. We document the morphological characteristics of various types of partial-thickness macular defects using spectral-domain optical coherence tomography, with the focus on tractional and degenerative lamellar holes, and discuss possible pathogenic mechanisms.

**Methods:**

A retrospective case series of 61 eyes of 61 patients with different types of partial-thickness macular defects is described.

**Results:**

Partial-thickness macular defects are caused by anteroposterior or tangential traction onto the fovea exerted by the partially detached posterior hyaloid and epiretinal membranes, respectively. Tractional elevation of the inner Müller cell layer of the foveola—without (outer lamellar holes, foveal pseudocysts) or with a disruption of this layer (tractional lamellar holes, macular pseudoholes)—produces an elevation of the inner layers of the foveal walls (nerve fiber layer to outer plexiform layer [OPL]) and a schisis between the OPL and Henle fiber layer (HFL). With the exception of outer lamellar holes, the (outer part of the) central outer nuclear layer and the external limiting membrane remain nondisrupted in the various types of partial-thickness defects. Degenerative lamellar holes are characterized by cavitations between the inner plexiform layer and HFL of the foveal walls; many cases have lamellar hole-associated epiretinal proliferation (LHEP). Proliferating cells of the disrupted Müller cell cone may contribute to the development of LHEP and fill the spaces left by degenerated photoreceptors in the foveal center.

**Conclusions:**

It is suggested that morphological characteristics of partial-thickness macular defects can be explained by the disruption of the (stalk of the) Müller cell cone in the foveola and the location of tissue layer interfaces with low mechanical stability: the boundary with no cellular connections between both Müller cell populations in the foveola, and the interface between the OPL and HFL in the foveal walls and parafovea. We propose that the development of the cavitations in degenerative lamellar holes is initiated by traction which produces a schisis between the OPL and HFL, and enlarged by a slow and chronic degeneration of Henle fibers and bipolar cells.

*Trial registration* retrospectively registered, #143/20-ek, 04/03/2020

## Background

The fovea is a pitted invagination in the inner retina which overlies an area of elongated thin photoreceptors. The foveal pit develops by a radial displacement of the inner retinal layers away from the path of the incoming light; this results in the formation of the central foveola surrounded by sloping foveal walls. The structural stability of the fovea is provided by Müller glia [1]. Two different populations of Müller cells are present in the fovea: (i) Specialized Müller cells in the foveola form the so-called Müller cell cone [2]. The horizontal layer of the Müller cell cone constitutes the inner layer of the foveola; the vertical stalk of the cone traverses the center of the foveola [1, 5]. The Müller cell cone provides critical structural support for the fovea and increases the resistance of the tissue against mechanical stress resulting from anteroposterior and tangential tractions which may occur, for example, in cystoid macular edema and after partial detachment of the posterior vitreous [1, 3, 5]. (ii) Müller cells of the foveal walls and parafovea have a characteristic z-shape because their outer processes run horizontally or obliquely through the Henle fiber layer (HFL) towards the foveal center; the Henle fibers, which are composed of photoreceptor axons surrounded by Müller cell processes, compensate the spatial shift between the inner and outer layers of the foveal tissue [4, 5]. The Müller cell cone also maintains the integrity of the foveal walls while the structural stability of the outer layers of the fovea is mainly provided by the Müller cells of the foveal walls [1].

Various macular diseases are associated with anteroposterior or tangential tractions exerted by contractile epiretinal membranes (ERM) and/or the partially detached posterior vitreous which may cause a disruption of the foveal integrity resulting in the formation of partial- or full-thickness macular defects. A full-thickness macular hole (FTMH) develops by disruptions of both the Müller cell cone and the external limiting membrane (ELM). The common feature of most types of partial-thickness macular defects is a tractional or degenerative disruption of the normal shape of the foveal pit; the (outer part of the) central outer nuclear layer (ONL) and the ELM are not disrupted and keep the foveal walls together which prevents the formation of a FTMH. Partial-thickness macular defects are grossly classified into macular pseudoholes, foveal pseudocysts, tractional lamellar holes, degenerative lamellar holes, and outer lamellar holes; mixed types of tractional and degenerative holes were also described [6–10]. Tractional lamellar holes are caused by traction exerted by contractile ERM or the partially detached posterior hyaloid and are mainly characterized by an intraretinal splitting (schisis) between the outer plexiform layer (OPL) and ONL of the foveal walls and parafovea [9, 10]. The main characteristic of degenerative lamellar holes is the development of cavitations of the foveal pit into the lower foveal walls [8–12]. Degenerative lamellar holes often show the development of a nonproliferative, nontractional, yellowish (macular pigment-containing) epiretinal tissue, termed lamellar macular hole-associated epiretinal proliferation (LHEP), above the nerve fiber layer (NFL) of the foveal walls and parafovea [9–11, 13–19]. The pathogenesis and functional role of this atypical epiretinal tissue are unclear. A certain number of cases of degenerative holes shows the presence of both tractional ERM and LHEP [20]. In spectral-domain optical coherence tomography (SD-OCT) scans, LHEP appears as a tissue of homogenous medium reflectivity which is lined by hyperreflective layers at the vitreal and retinal surfaces of this tissue. Various cell types were suggested to contribute to the formation of LHEP, including lymphocytes, fibroblasts, hyalocytes, retinal pigment epithelial cells, and macular pigment-containing glial cells [14, 15, 17, 21].

The aims of the present study were (i) to describe the morphological OCT characteristics of various types of partial-thickness macular defects, with the focus on the comparison of the morphologies of tractional and degenerative lamellar holes, and (ii) to discuss possible mechanisms of the pathogenesis of tractional and degenerative lamellar holes, including the roles of Müller cells and of tissue layer interfaces of low mechanical stability in the pathological processes. Because other types of partial-thickness defects may also display a schisis between the OPL and ONL of the foveal walls [22], we included cases of tractionally induced foveal pseudocysts, outer lamellar holes, and macular pseudoholes.

## Methods

This is a retrospective, single-center chart review. The study followed the ethical standards of the 1964 Declaration of Helsinki and its later amendments. The protocol was approved by the Ethics Committee of the Medical Faculty of the University of Leipzig (#143/20-ek, 04/03/2020). The ethics committee is registered as Institutional Review Board at the Office for Human Research Protections (registration number, IORG0001320/IRB00001750). We retrospectively reviewed charts of patients with partial-thickness macular defects who were referred to the Department of Ophthalmology, University of Leipzig, Germany, between February 2009 and August 2019. Patients with a lamellar macular hole or other types of tractionally induced structural alterations of the fovea in at least one eye were included in the study. Exclusion criteria were FTMH (with the exception of one case of a FTMH with LHEP) and degenerative myopia, defined as axial length of > 26 mm with presence of a pathological myopic maculopathy. Cross-sectional images of the macular area were obtained with SD-OCT (Spectralis, Heidelberg Engineering, Heidelberg, Germany). Best-corrected visual acuity (BCVA) was determined with a Snellen chart and is given in decimal units.

**Fig. 1:**
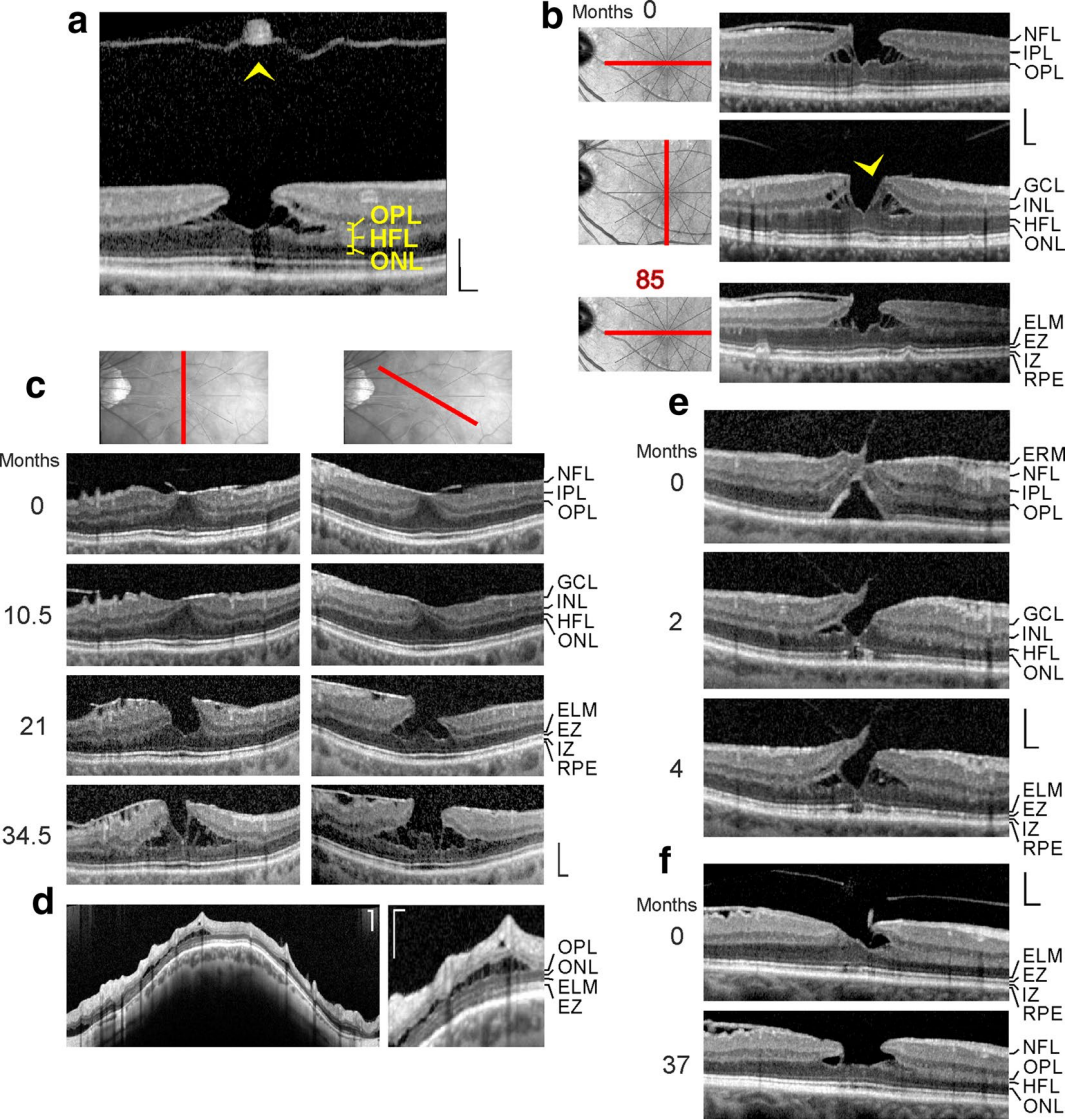
Schistic splitting of the foveal walls between the outer plexiform layer (OPL) and Henle fiber layer (HFL) as a characteristic of tractional lamellar holes. The images show SD-OCT scans through the fovea and parafovea of 5 eyes of 5 patients. In **c**, **e**, **f** the months after the first visit (0) are indicated left of the images. **a** The presence of an operculum (arrowhead) may suggest that traction exerted by the posterior hyaloid removed the inner Müller cell layer from the foveola. The tissue of the foveal walls splitted between the OPL and HFL. **b** The scans were recorded at and 85 months after the first visit. The orientations of the scans are shown at the left side. The arrowhead indicates a tissue band with medium reflectivity extending from the center of the foveola to the edge of the elevated dorsal foveal wall. **c** Development of a tractional lamellar hole from a macular pseudohole. The orientations of the scans are shown above. Note the presence of epiretinal membranes (ERM) which bridged deep retinal folds in the fovea and parafovea. Note also that the fovea externa was not disrupted. **d** Circumpapillary scans recorded in the same eye 34.5 months after the first visit. The right image shows a part of the image at higher magnification. Note the schistic splitting between the OPL and ONL at various sites of the peripapillary retina which was associated with a disappearance of the ellipsoid zone (EZ) line. **e** Development of a lamellar hole by tractional detachment of the foveola from the retinal pigment epithelium (RPE) and subsequent disruption of the junction between the inner layer of the foveola and the nasal foveal wall. The disruption allowed a reattachment of the central outer nuclear layer (ONL) at the RPE and was associated with a schistic splitting of the foveal walls between the OPL and HFL. An ERM was present in the nasal parafovea. **f** Development of a tractional lamellar hole after disruption of the Müller cell cone. Scale bars, 200 µm. ELM: external limiting membrane; GCL: ganglion cell layer; ELM: external limiting membrane; INL: inner nuclear layer; IPL: inner plexiform layer; IZ: interdigitation zone; NFL: nerve fiber layer

**Fig. 2:**
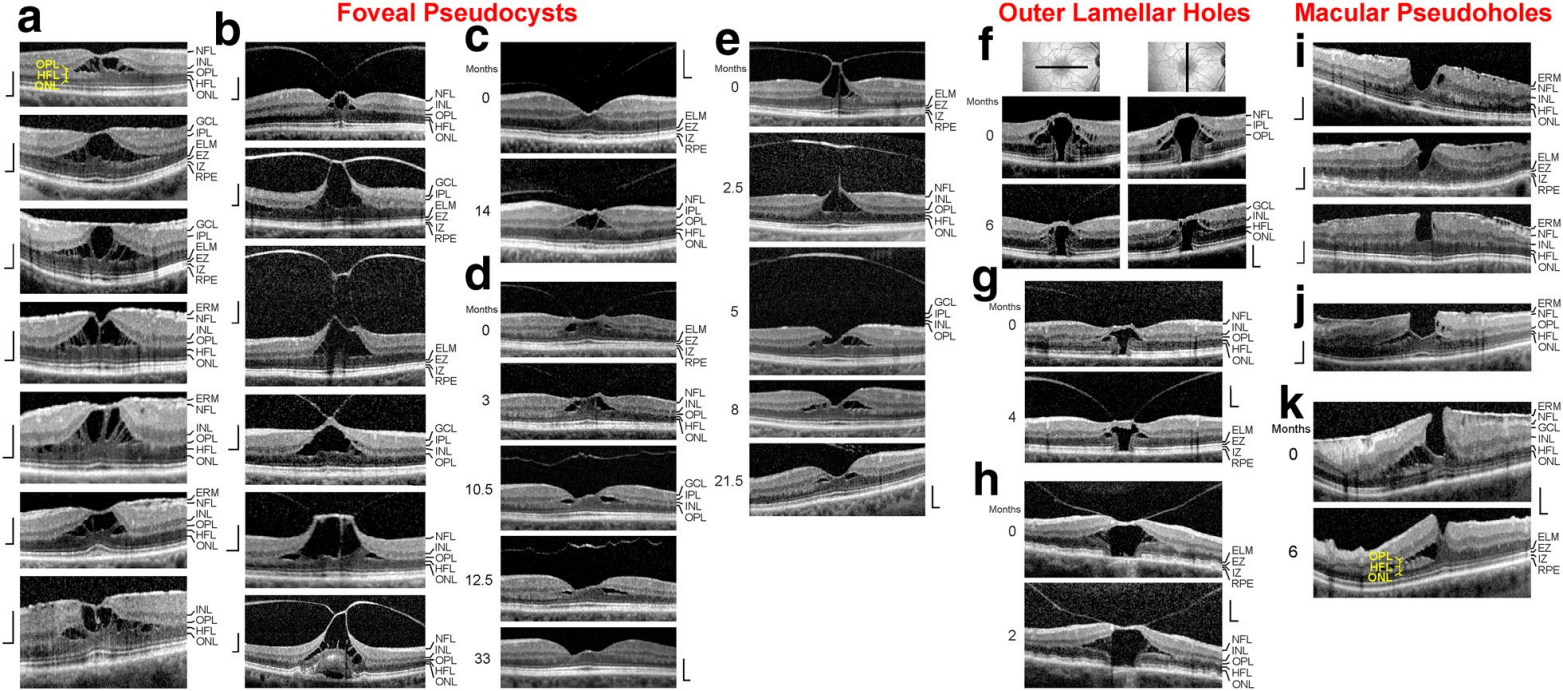
Schistic splitting of the foveal walls between the outer plexiform layer (OPL) and Henle fiber layer (HFL) in foveal pseudocysts (**a**‒**e**), outer lamellar holes (**f**–**h**), and macular pseudoholes (**j**, **k**). The images show linear SD-OCT scans through the fovea and parafovea of 24 eyes of 24 patients. **a** Foveal pseudocysts which were likely produced by tangential traction exerted by epiretinal membranes (ERM). **b**‒**e** Foveal pseudocysts which were produced by anteroposterior traction exerted by the partially detached posterior hyaloid. **c** Tractional development of a foveal pseudocyst. The months after the first visit (0) are indicated left of the images. Note the hyperreflectivity of the inner Müller cell layer of the foveola. **d**, **e** Regeneration of the foveal shape after relief of the vitreofoveal traction. **f**‒**h** The outer lamellar holes were produced by vitreomacular traction exerted by the posterior hyaloid attached to the foveola. In **f**, the orientations of the scans are shown above. **i**‒**k** Macular pseudoholes without (**i**) and with (**j**, **k**) a schistic splitting of the foveal walls. The scans in **k** were recorded at the first visit (0) and 6 months later. Pars plana vitrectomy with internal limiting membrane and ERM peeling was performed 2.5 months after the first visit. Scale bars, 200 µm. ELM: external limiting membrane; EZ: ellipsoid zone; GCL: ganglion cell layer; INL: inner nuclear layer; IPL: inner plexiform layer; IZ: interdigitation zone; NFL: nerve fiber layer; ONL: outer nuclear layer; RPE: retinal pigment epithelium

**Fig. 3:**
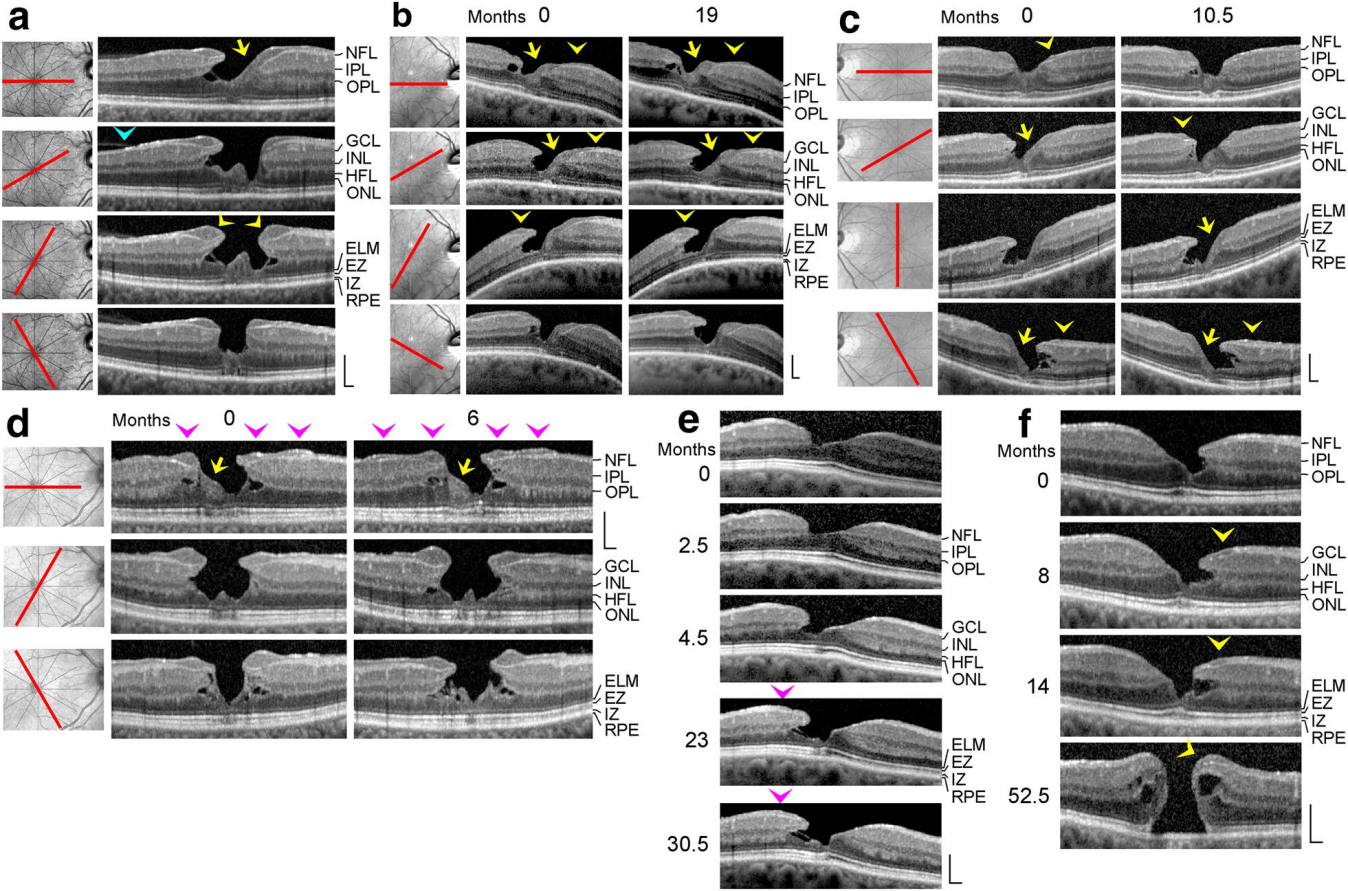
Cavitations of the foveal pit in the lower foveal walls as a characteristic of degenerative lamellar macular holes in 6 eyes of 6 patients. The months after the first visit (0) are indicated above or left of the images. Yellow and pink arrowheads indicate lamellar hole-associated epiretinal proliferation (LHEP). The arrows indicate tissue bands of medium reflectivity which connect glial cells in the center of the foveola with LHEP at the inner surface of the foveal walls. **a**–**d** Radial scans of four cases of a degenerative lamellar macular hole. The orientations of the SD-OCT scans are shown at the left side. The blue arrowhead in **a** indicates the adherence of a membrane to the temporal parafovea. **e** Development of a degenerative lamellar hole. **f** Development of a full-thickness macular hole from a lamellar hole. Scale bars, 200 µm. ELM, external limiting membrane; EZ, ellipsoid zone; GCL, ganglion cell layer; HFL, Henle fiber layer; INL, inner nuclear layer; IPL, inner plexiform layer; IZ, interdigitation zone; NFL, nerve fiber layer; ONL, outer nuclear layer; OPL, outer plexiform layer; RPE, retinal pigment epithelium

**Fig. 4:**
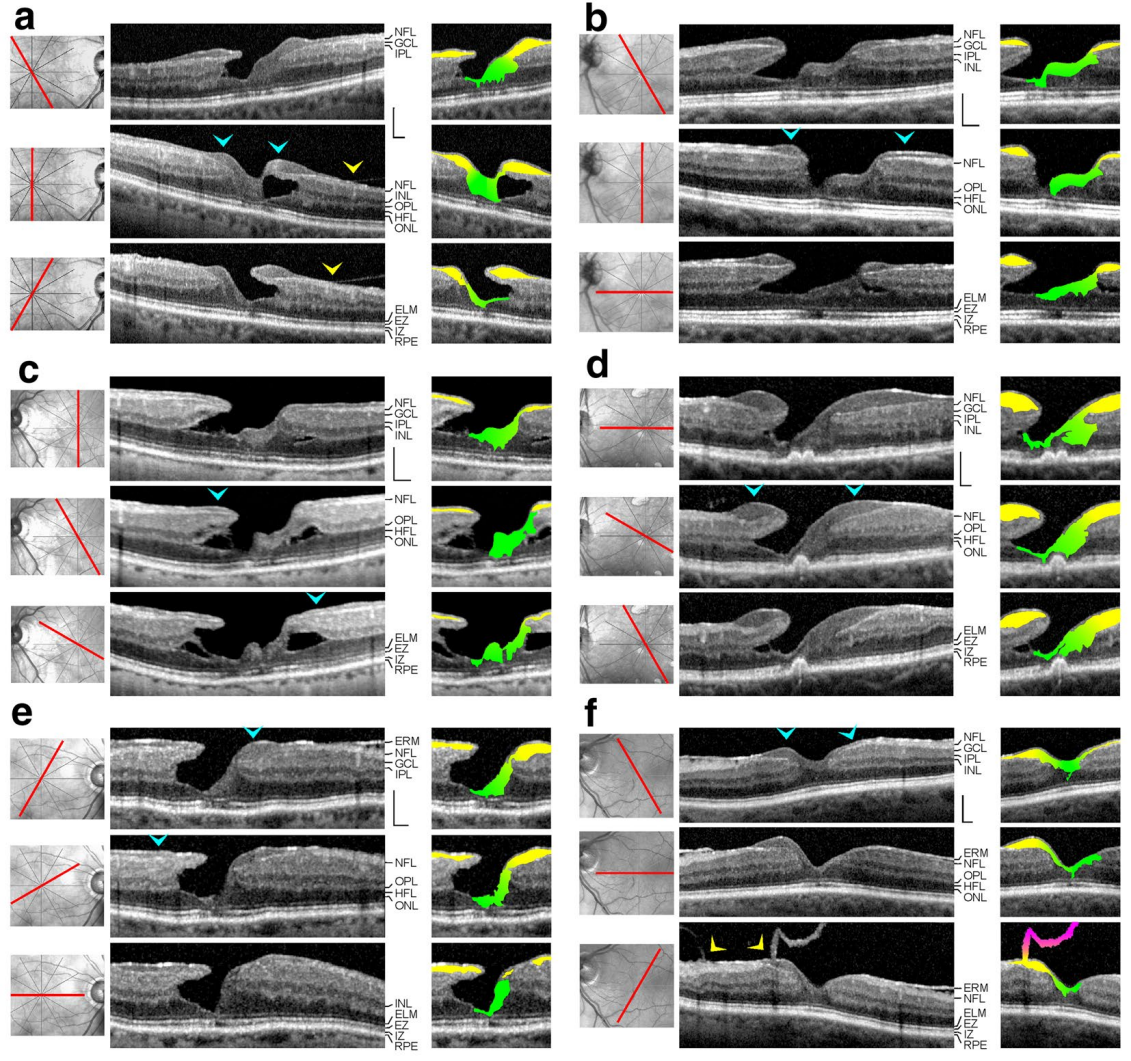
In degenerative lamellar holes, Müller cells in the foveola are connected to the middle part of lamellar macular hole-associated epiretinal proliferation (LHEP) at the inner surface of the foveal walls. The images show SD-OCT scans through the fovea and parafovea of 6 eyes of 6 patients. The orientations of the scans are shown at the left side. The blue arrowheads indicate LHEP. The yellow arrowheads indicate vitreomacular adhesions. In the smaller images at the right side, the middle part of LHEP is indicated by yellow color, cells of the Müller cell cone are indicated by green color, and the posterior hyaloid is indicated by pink color. Scale bars, 200 µm. ELM, external limiting membrane; ERM: epiretinal membrane; EZ: ellipsoid zone; GCL: ganglion cell layer; HFL: Henle fiber layer; INL: inner nuclear layer; IPL: inner plexiform layer; IZ: interdigitation zone; NFL: nerve fiber layer; ONL: outer nuclear layer; OPL: outer plexiform layer; RPE: retinal pigment epithelium

**Fig. 5:**
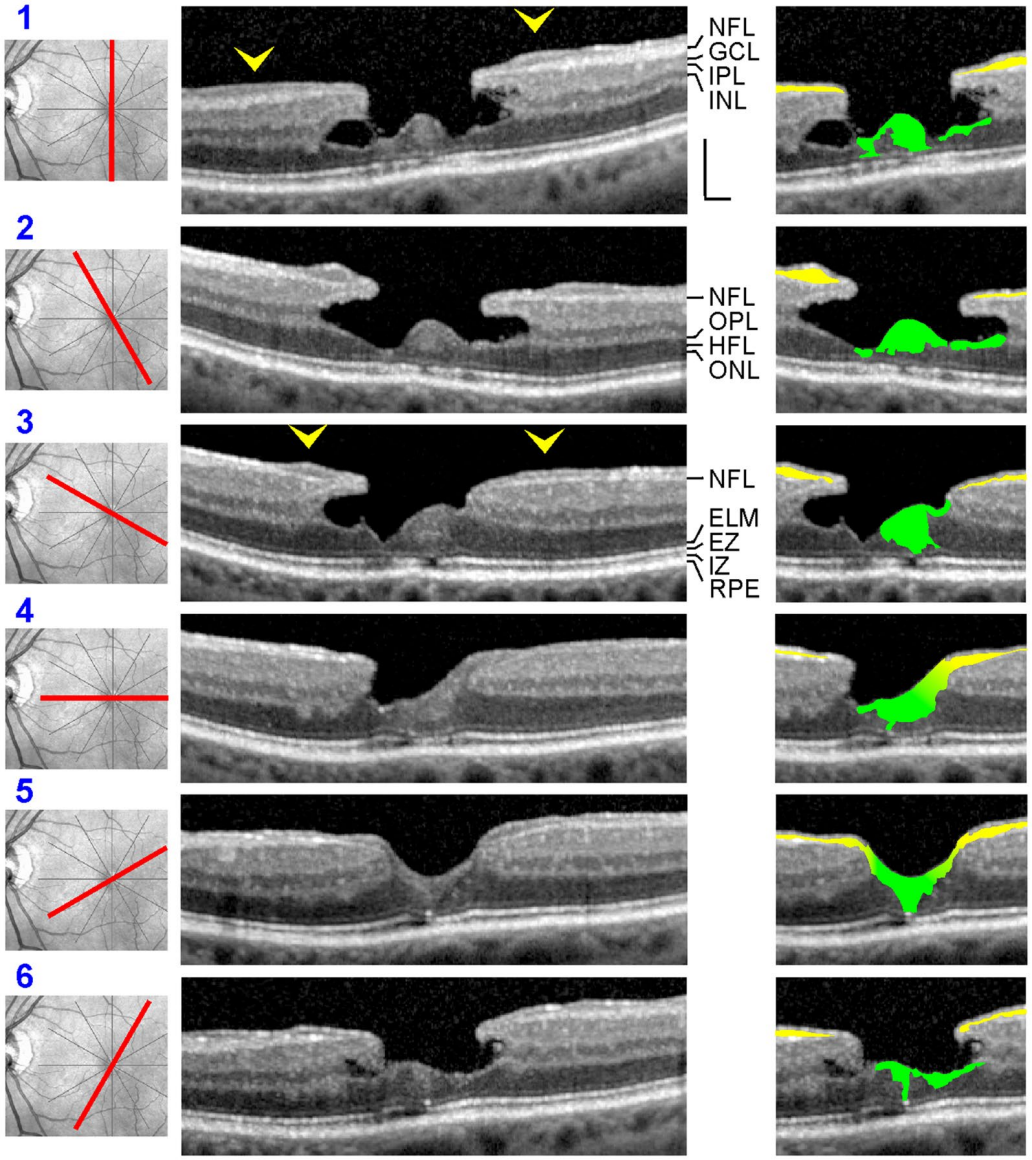
The connection between Müller cells in the foveola and the middle part of lamellar macular hole-associated epiretinal proliferation (LHEP) at the inner surface of the foveal walls is visible only in certain orientations of SD-OCT scans. The images show radial scans through the fovea and parafovea in the left eye of a 77 year-old man. The orientations of the scans are shown at the left side. The arrowheads indicate LHEP. In the smaller images at the right side, the middle part of LHEP is indicated by yellow color, and cells of the Müller cell cone are indicated by green color. Note that there are no connections between Müller cells in the foveola and LHEP in the scans which show cavitations of the foveal pit into the lower foveal walls (scans 1‒3 and 6) whereas in the scans 4 and 5, foveal walls without cavitations display such connections. Scale bars, 200 µm. ELM: external limiting membrane; EZ: ellipsoid zone; GCL: ganglion cell layer; HFL: Henle fiber layer; INL: inner nuclear layer; IPL: inner plexiform layer; IZ: interdigitation zone; NFL: nerve fiber layer; ONL: outer nuclear layer; OPL: outer plexiform layer; RPE: retinal pigment epithelium

**Fig. 6:**
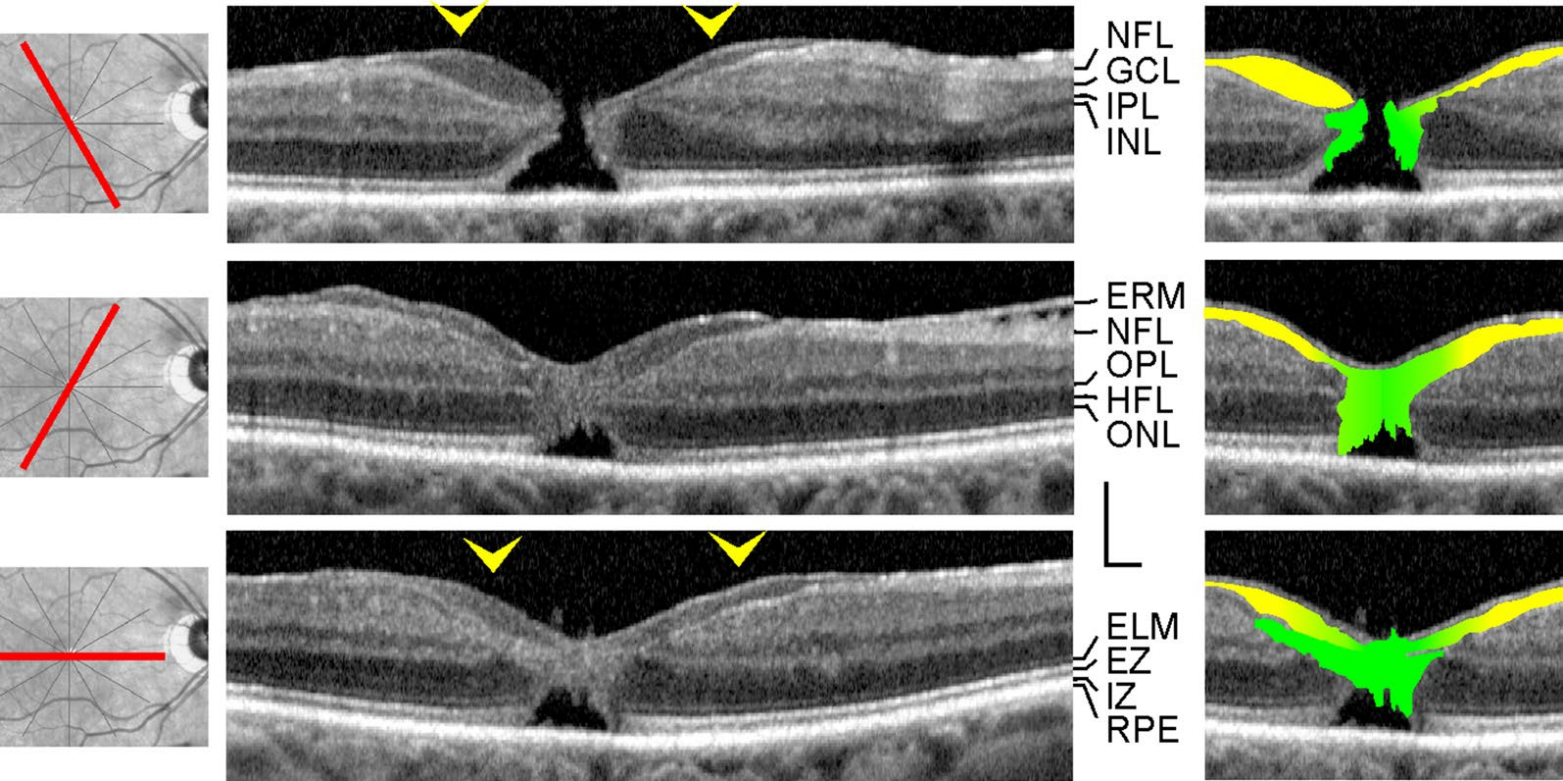
Example of a full-thickness macular hole with lamellar macular hole-associated epiretinal proliferation (LHEP) at the inner surface of the foveal walls. The images show SD-OCT scans through the fovea and parafovea of the right eye of a 76 year-old man. The orientations of the scans are shown at the left side. The arrowheads indicate LHEP. In the smaller images at the right side, the middle part of LHEP is indicated by yellow color, and cells of the Müller cell cone are indicated by green color. Scale bars, 200 µm. ELM: external limiting membrane; ERM: epiretinal membrane; EZ: ellipsoid zone; GCL: ganglion cell layer; HFL: Henle fiber layer; INL: inner nuclear layer; IPL: inner plexiform layer; IZ: interdigitation zone; NFL: nerve fiber layer; ONL: outer nuclear layer; OPL: outer plexiform layer; RPE: retinal pigment epithelium

**Fig. 7:**
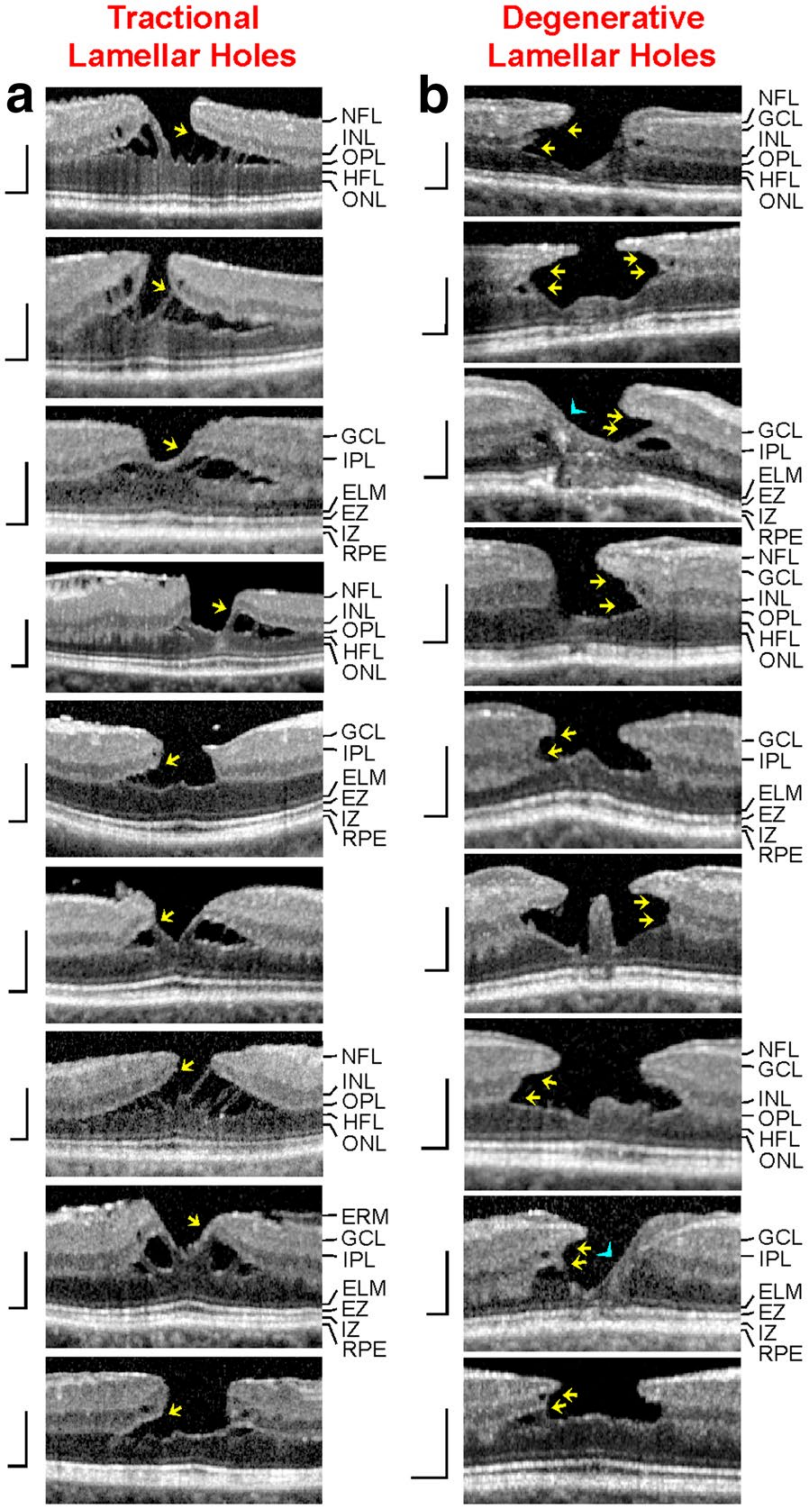
Comparison of schistic and degenerative cavitations in tractional and degenerative lamellar holes, respectively. The images show linear SD-OCT scans through the fovea of 18 eyes of 18 patients. **a** Tractional lamellar holes. **b** Degenerative lamellar holes. The arrows indicate the levels of the widest lateral extensions of schistic (**a**) and degenerative cavitations (**b**). The arrowheads indicate morphological connections between Müller cells in the foveola and lamellar macular hole-associated epiretinal proliferation. Scale bars, 200 µm. ELM: external limiting membrane; ERM: epiretinal membrane; EZ: ellipsoid zone; GCL: ganglion cell layer; HFL: Henle fiber layer; INL: inner nuclear layer; IPL: inner plexiform layer; IZ: interdigitation zone; NFL: nerve fiber layer; ONL: outer nuclear layer; OPL: outer plexiform layer; RPE: retinal pigment epithelium

All patients were Caucasians. SD-OCT images of the macular region of one eye of 14 patients with a tractional lamellar hole were investigated (Figs. 1a‒f and 7a; 12 women, 2 men; mean ± S.D. age, 67.2 ± 8.9 years; range 45‒76 years). The mean BCVA was 0.71 ± 0.26 (range, 0.10‒1.00). SD-OCT scans of the macular region of one eye of 16 further women (mean age, 65.8 ± 9.5 years; range 45‒77 years; mean BCVA 0.60 ± 0.30, range, 0.12‒1.00) showed foveal pseudocysts (Fig. 2a‒e). Three women with an outer lamellar hole in one eye were investigated (Fig. 2f‒h; mean age, 71.3 ± 13.0 years, range 61‒86 years); the BCVA ranged from 0.35 to 0.80 (mean, 0.58 ± 0.22). SD-OCT images recorded in one eye of 5 patients revealed the presence of a macular pseudohole (Fig. 2i‒K; 3 women, 2 men; mean age, 70.6 ± 7.2 years, range 59‒77 years; mean BCVA 0.55 ± 0.18, range, 0.32‒0.80). Twenty-two patients with a degenerative lamellar hole in one eye were investigated (Figs. 3a‒f, 4a‒f, 5, and 7b; 10 women, 12 men; mean age, 72.7 ± 7.9 years, range 52‒87 years; mean BCVA 0.66 ± 0.16 range, 0.30‒0.90). In addition, one eye of a 76 year-old man with a FTMH was investigated (Fig. 6; BCVA, 0.7).

## Results

### Schistic tissue splitting in tractional lamellar holes

Tractional lamellar holes are characterized by a disruption of the Müller cell cone in the foveola and an elevation of the inner layers of the foveal walls (NFL to OPL) which causes a schistic splitting of the foveal walls and parafovea between the OPL and HFL; obliquely arranged bundles of Henle fibers traverse the schistic cavities (Fig. 1a, b). Normally, the central ONL and the hyperreflective lines in the outer retina display no abnormalities. The presence of an operculum at the posterior hyaloid in the case shown in Fig. 1a may suggest that anteroposterior traction removed a part of the inner Müller cell layer from the foveola and caused the elevation of the inner layers of the foveal walls. The lamellar hole shown in Fig. 1b was likely produced by traction from both an ERM attached at the nasal foveal wall and the posterior hyaloid which adhered at the dorsal and ventral foveal walls.

It was described that a tractional lamellar hole can develop from a macular pseudohole [22]. This was observed in the case shown in Fig. 1c. SD-OCT scans recorded at the first visit showed the presence of ERM which bridged deep retinal folds at the inner surface of the parafovea and that caused elevations of the foveola and the inner layers of the foveal walls, resulting in a thickening of the central ONL and a flattening of the foveal pit. Between 10.5 and 21 months after the first examination, a pseudohole developed. Thereafter, tangential traction exerted by ERM caused a further elevation of the inner layers of the foveal walls which was associated with a schistic tissue splitting between the OPL and HFL. Circumpapillary scans recorded in this eye showed a schistic splitting between the OPL and ONL at various sites of the peripapillary retina which was associated with a disapperance of the ellipsoid zone (EZ) line (Fig. 1d).

The lamellar hole shown in Fig. 1e was created by anteroposterior traction exerted by the posterior hyaloid. The traction caused a detachment of the whole foveola from the retinal pigment epithelium (RPE). Within 2 months after the first examination, the traction produced a disruption of the junction between the inner layer of the foveola and the nasal foveal wall. This was associated with a reattachment of the central ONL at the RPE; this and the remaining elevation of the inner layers of the walls caused a schistic splitting of the foveal walls between the OPL and HFL. Figure 1f shows the development of a tractional lamellar hole after disruption of the Müller cell cone.

### Schistic tissue splitting in other types of partial-thickness macular defects

Foveal pseudocysts are characterized by a tractional disruption of the foveal structure resulting in cyst formation in the foveola and foveal walls; the traction is exerted by contractile ERM (Fig. 2a) and/or the partially detached posterior hyaloid (Fig. 2b‒e). The morphology of foveal pseudocysts is similar to that of tractional lamellar holes with the exception that the horizontal layer of the Müller cell cone, which is detached from the central HFL/ONL resulting in a stretching or disruption of the stalk of the cone, is not disrupted and keeps the elevated inner layers of the foveal walls together (Fig. 2a‒c). As in tractional lamellar holes, there is a schistic splitting of the foveal walls between the OPL and HFL while, in most cases, the central ONL and photoreceptor layer remain nonaffected. The extent of the elevation of the inner layers of the foveal walls is one factor which determines the size of the schisis between the OPL and HFL.

In the cases shown in Fig. 2d and e, anteroposterior traction produced a foveal pseudocyst associated with a schistic splitting of the foveal walls between the OPL and HFL; after relief of the traction, the pseudocysts disappeared and the size of the schistic tissue splitting decreased time-dependently. The finding that the size of the schistic tissue splitting decreased after relief of the traction and the regeneration of the Müller cell cone (Fig. 2e) may suggest that a nondisrupted Müller cell cone is required for the prevention of a schistic splitting of the foveal walls.

Figure 2f–h shows examples of outer lamellar holes. Outer lamellar holes are characterized by a central pseudocyst produced by a detachment of the inner layer of the foveola from the HFL/ONL due to tractional forces exerted by the posterior hyaloid. The elevation of the inner layer of the foveola produces an elevation of the inner layers of the foveal walls (NFL to OPL) which causes an oblique stretching and straightening of the Müller cells of the foveal walls that transmit the tension to the outer retina. This produces a centrifugal displacement of the central ONL and photoreceptors resulting in a disruption of the ELM and a hole in the outer retina [6, 23]. The elevation of the inner layers of the foveal walls was accompanied by a schistic tissue splitting between the OPL and HFL, and cystic cavities in the inner nuclear layer (INL). The size of the schistic tissue splitting varied with the extent of the elevation of the inner layers of the foveal walls (Fig. 2f).

Macular pseudoholes are produced by traction exerted by contractile ERM which causes a thickening of the foveal tissue. Many cases of macular pseudoholes do not show a schistic splitting of the foveal walls (Fig. 2i); however, there are also cases with a schisis between the OPL and HFL (Fig. 2j, k) [22]. In the case shown in Fig. 2k, the traction produced both a thickening of the foveal tissue and an elevation of the inner layers of the foveal walls. After pars plana vitrectomy with internal limiting membrane and ERM peeling, the size of the schistic tissue splitting decreased.

### Degenerative lamellar holes

Degenerative lamellar holes are characterized by cavitations of the foveal pit into the lower foveal walls; the foveal walls above the cavitations are elevated (Fig. 3a‒f). The development of the degenerative cavitations is often associated with central photoreceptor layer defects [9, 20]. Many cases of degenerative lamellar holes also show LHEP at the vitreal surface of the foveal walls and parafovea (Fig. 3a‒f). LHEP is composed of a tissue of medium reflectivity and hyperreflective layers at the vitreal and retinal sides of this tissue.

The SD-OCT scans shown in Fig. 3a‒d display various characteristics of a degenerative lamellar hole: large cavitations of the foveal pit into the lower foveal walls, LHEP at the vitreal surface of the foveal walls, and a loss of the integrity of the central photoreceptor segments, as indicated by the central defects of the ELM, EZ, and interdigitation zone (IZ) lines. In addition, there were tissue disruptions between the OPL and IPL in the foveal walls (Fig. 3b‒d). In the cases shown in Fig. 3a and d, the degenerative cavitations were connected to cystic cavities between the OPL and HFL of the foveal walls; obliquely arranged thin bundles of Henle fibers bridged these cavities. The centers of the foveas were partly devoid of the ONL; these parts were filled by a tissue of medium reflectivity. The medium reflectivity suggests that this tissue was formed by Müller cells, and the location suggests that it was formed by hypertrophied and/or proliferating cells of the Müller cell cone. There were broad tissue bands of medium reflectivity which covered the whole inner surface of nonelevated foveal walls and that connected the cells of the Müller cell cone in the foveola with LHEP at the vitreal surface of the walls (arrows in Fig. 3a‒d). Furthermore, the fovea externa, i.e., the cone-like arrangement of the elongated central photoreceptor segments, was irregularly formed with the tip directed to the RPE and not to the vitreous as in the normal fovea (Fig. 3a‒d). The outward deflections of the ELM and EZ lines were spatially associated with the tissues of medium reflectivity which filled the ONL-free parts of the foveola.

Figure 3e shows the early unilateral development of a degenerative lamellar hole. Apparently, tractional forces exerted by contraction of an ERM which lied at the NFL of one foveal wall caused an alteration of the foveal contour; this likely resulted from a disruption of the connection between the inner Müller cell layer of the foveola to this wall which produced an indentation of the foveal pit between the OPL and HFL (first visit and 2.5 months later). In the further course (4.5 months), this indentation developed to a schisis which was associated with a thinning of the HFL/ONL in this part of the foveola and a loss of the integrity of the central photoreceptors, as indicated by the hyporeflectivities of the EZ and IZ lines. Thereafter, the schisis developed to a degenerative cavitation which was traversed by thin bundles of Henle fibers (23 and 30.5 months). Along with the development of the degenerative cavitation, LHEP appeared at the foveal wall and parafovea. Between 23 and 30.5 months, there was an enlargement of a tissue with medium reflectivity at the inner side of the foveola, likely representing proliferating cells of the Müller cell cone.

Figure 3f shows a degenerative lamellar hole which developed to a FTMH. The parafovea displayed hyperreflective innermost layers indicating the presence of ERM. The morphology of the lamellar hole altered little until 14 months after the first examination, with the exception of the development of LHEP at the inner surface of the lower foveal wall. Thereafter, a FTMH developed; the inner layers of the foveal walls around the hole were strongly elevated, and the foveal walls contained large cystic cavities.

### Morphological relation between foveolar Müller cells and LHEP

It was described that LHEP at the inner surface of the foveal walls may be connected with Müller cells in the foveola [11, 16, 21]. In the cases shown in Fig. 4a‒e, the tissues of medium reflectivity in the foveola, likely formed by Müller cells, were connected to the middle part of LHEP which also displayed a medium reflectivity. The connecting tissues between the Müller cells in the foveola and LHEP were preferentially present in such parts of the fovea which did not contain degenerative cavitations of the foveal pit into the lower foveal walls (Figs. 4a‒e and 5). In parts of the fovea in which the inner layers of the foveal walls were elevated above degenerative cavitations, there were regularly no connections between the Müller cells in the foveola and LHEP, with some exceptions like the dorsal foveal wall in the case shown in Fig. 4a, the temporal foveal wall in the case shown in Fig. 4b, and the ventrotemporal foveal wall in the case shown in Fig. 4c. In these cases, the connections formed tissue bridges which spanned the cavitations. The presence of “humps” in the center of the foveola which are often visible in SD-OCT images of degenerative lamellar holes (e.g., Figs. 3a and 4c, and scans 1 and 2 in Fig. 5) can be explained with the fact that foveolar Müller cells are connected only to certain foveal walls. A similar connection between foveolar Müller cells and LHEP was found in a case of a FTMH (Fig. 6). In this case, the foveal walls did not contain cystic cavities, and the inner layers of the walls were not elevated.

### Morphological relation between ERM and LHEP

As previously shown [15, 20], ERM are mainly present eccentric from the fovea while LHEP are preferentially located at the foveal edges (Figs. 4e, f, and 6). In the cases examined in the present study, ERM continued into the inner hyperreflective layer of LHEP (Fig. 6) or into both hyperreflective layers of LHEP (Fig. 4f). In the case of the FTMH shown in Fig. 6, there was a continuity between the ERM at the dorsonasal parafovea and the inner hyperreflective layer of LHEP. The hyperreflective layer at the retinal side of LHEP is often formed by the hyperreflective NFL (e.g., Fig. 3a).

The fovea shown in Fig. 4f had a nearly normal shape with the exceptions of the presence of LHEP at the foveal walls and a relatively thick tissue of medium reflectivity which filled the inner part of the foveola. This tissue, likely formed by Müller cells, continued to the middle part of LHEP which also displayed a medium reflectivity. There were ERM at the inner surfaces of the dorsonasal and temporal foveal walls; the ERM in the nasal parafovea continued to the inner and outer hyperreflective layers of LHEP. In addition, there were vitreomacular adhesions at the inferior parafovea; the tissue of the thicker vitreous remnant continued to the middle part of LHEP.

### Comparison of tractional and degenerative lamellar holes

Both degenerative and tractional lamellar holes are likely produced by a tractional disruption of the Müller cell cone and an elevation of the inner layers of the foveal walls. However, the elevation of the inner layers of the foveal walls has partially different consequences on the contours of the cavities in the foveal walls in both types of lamellar holes. In most cases of tractional holes, the elevated inner layers of the foveal walls included the NFL to OPL; the elevation resulted in a schisis between the OPL and HFL (Fig. 7a). The schisis is tapered, has a more regular contour, and is traversed by relatively thick bundles of Henle fibers. Most cases of tractional lamellar holes displayed no defects of the central ONL and photoreceptor layer (Fig. 7a).

In degenerative lamellar holes, the shape of the cavitations of the foveal pit into the lower foveal walls varied among the individuals. Among the cases presented in Fig. 7b, the widest lateral extension of the cavitations was either at the interface between the inner plexiform layer (IPL) and INL or at the OPL-HFL interface; the latter resembles the location of the schisis observed in tractional lamellar holes. Cavitations with the widest extension at the OPL-HFL interface had a second step of extension at the IPL-INL interface. The outer layer of the elevated foveal walls, which protruded centripetally above the degenerative cavitations, was the OPL and, more centrally, the IPL. The degenerative cavitations were not traversed by bundles of Henle fibers or were traversed by thin bundles (Fig. 7b). In most cases of degenerative lamellar holes, the central ONL and photoreceptor layer showed degenerative alterations (Fig. 7b). An exception was the case shown at the bottom of Fig. 7b which likely represents a mixed type of lamellar holes without LHEP and no apparent degeneration of the central outer retina.

## Discussion

**Fig. 8:**
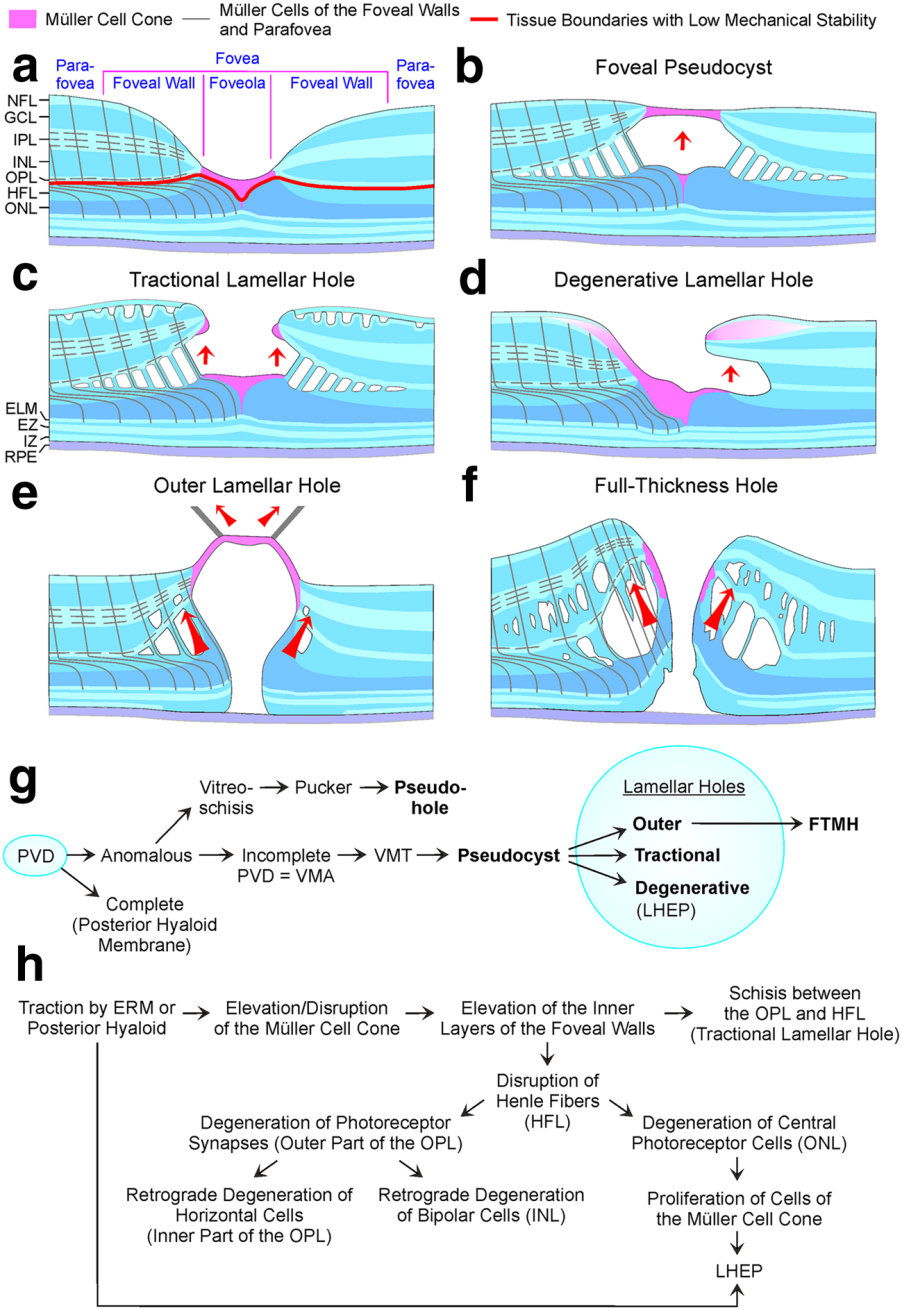
Hypothetical mechanisms of the development of partial- and full-thickness macular defects. **a** Schematic section through a fovea. The Müller cell cone in the foveola is shown in pink. The tissue layer interfaces of low mechanical stability are indicated by red lines: the boundary between the Müller cell cone and the Henle fiber layer (HFL)/outer nuclear layer (ONL) in the foveola, and the interface between the outer plexiform layer (OPL) and HFL in the foveal walls and parafovea. In addition, the vertical stalk of the Müller cell cone in the center of the foveola has a low mechanical stability. **b** The horizontal layer of the Müller cell cone keeps the inner layers of the foveal walls (nerve fiber layer [NFL] to OPL) together. Normally, the stalk of the Müller cell cone prevents the elevation of the inner layers of the foveal walls. When anteroposterior or tangential tractions exerted by the posterior hyaloid or epiretinal membranes (ERM) disrupt the stalk, foveal pseudocysts associated with an elevation of the inner layers of the foveal walls may develop. The elevation of the inner layers of the foveal walls disrupts the tissue between the OPL and HFL resulting in the formation of schistic cavities which are obliquely traversed by Henle fiber bundles. **c**, **d** Anteroposterior or tangential traction may cause a disruption of the connection between the Müller cell cone and the foveal walls, resulting in an elevation of the inner layers of the walls. This may produce a schisis between the OPL and HFL in the foveal walls (**c**) which may develop to degenerative cavitations of the foveal pit into the lower foveal walls (**d**). Bundles of Henle fibers composed of photoreceptor axons and the outer processes of Müller cells of the foveal walls keep the schistic cavities together (**c**). The degenerative cavitations may be enlarged by a degeneration of Henle fibers (**d**). The formation of a degenerative lamellar hole is often associated with a disruption of the fovea externa (**d**). Macular pigment-containing cells of the Müller cell cone may contribute to the development of the lamellar hole-associated epiretinal proliferation (LHEP), likely by the formation of a tissue bridge between the foveola and the inner surface of the foveal walls (**d**). **e** Anteroposterior traction may cause the formation of an outer lamellar hole characterized by a large pseudocyst in the foveola, schistic splitting of the foveal walls between the OPL and HFL, cystic cavities in the inner nuclear layer (INL), and a gap in the whole central outer retina including the ELM. A disruption of the Müller cell cone produces a full-thickness macular hole (FTMH) from an outer lamellar hole. **f** Fluid accumulation in the foveal walls may produce edematous cysts between the OPL and HFL, and in the INL. Enlargement of the cysts causes a large elevation of the inner layers of the walls; the Müller cells are obliquely stretched and straightened, and transmit the tension to the outer retina. This produces a detachment and a centrifugal displacement of the central ONL and photoreceptors resulting in an enlargement of the FTMH. **g** Schematic summary of pathogenic steps which mediate the development of partial-thickness macular defects and FTMH. **h** Pathogenic events which may be implicated in the development of degenerative cavitations of the foveal pit into the lower foveal walls. EZ: ellipsoid zone; GCL: ganglion cell layer; IPL: inner plexiform layer; IZ: interdigitation zone; NFL: nerve fiber layer; PVD: posterior vitreous detachment; RPE: retinal pigment epithelium; VMA: vitreomacular adhesion; VMT: vitreomacular traction

Most types of partial-thickness macular defects are characterized by a deformation of the foveal pit due the tractional elevation or disruption of the Müller cell cone while the (outer part of the) central ONL and the ELM remain unaffected and keep the foveal walls together (Fig. 8b‒d). In one type, the outer lamellar hole, the central ONL and the ELM are disrupted (Fig. 8e) [6]. A FTMH often develops from a foveal pseudocyst and an outer lamellar hole after the disruption of the Müller cell cone (Fig. 8f) [7, 24, 25]. Figure 8g shows a schematic summary of pathogenic steps which may lead to the development of partial- and full-thickness macular defects.

### Location of schistic and degenerative cavitations in lamellar holes

We propose that certain morphological characteristics of partial-thickness macular defects can be explained with the features of both Müller cell populations in the fovea and the localization of tissue layer interfaces with low mechanical stability: the boundary between the Müller cell cone and the HFL/ONL in the foveola, and the interface between the OPL and HFL in the foveal walls and parafovea (Fig. 8a). The low mechanical stability of the boundary between the Müller cell cone and the HFL/ONL in the foveola results from the absence of cellular connections between the cells of the Müller cell cone and the outer processes of the Müller cells of the foveal walls which envelop the somata and fibers of the photoreceptor cells in the HFL/ONL [1, 5]. This and the low stability of the vertical stalk of the Müller cell cone in the center of the foveola, which easily disrupts in the presence of tractional forces onto the fovea [1], are the reasons why traction can detach (like in foveal pseudocysts; Fig. 2A‒E) or remove parts of the inner Müller cell layer of the foveola from the underlying HFL/ONL (Fig. 1a). A disruption of the Müller cell cone or of the connection between the Müller cell cone and the foveal walls seems to be one pathogenic event involved in the development of certain morphological features of partial-thickness defects. The disruption is caused by tractional forces which evolve from contractile ERM and/or the partially detached posterior hyaloid [24–27].

Under normal conditions, the structural stability of the fovea is provided by both Müller cell populations, i.e., the cells of the Müller cell cone and Müller cells of the foveal walls (Fig. 8a) [1]. The finding that in most types of partial-thickness defects the outer part of the central ONL and the ELM are not disrupted and keep the foveal walls together (Fig. 8b‒d) can be explained with the fact that these structures are not stabilized by the Müller cell cone, but by the outer processes of the Müller cells of the foveal walls; these processes draw from the OPL through the HFL towards the foveal center, enclose the fibers and somata of photoreceptor cells, and constitute, together with the photoreceptor cells, the ELM (Fig. 8a) [1, 4, 5]. Photoreceptor and Müller cells are tightly glued together in the HFL and ONL, and at the ELM [28, 29]. At the ELM, Müller cells contain contractile rings of filamentous actin which enclose the photoreceptors; these rings are associated with the junctions between Müller and photoreceptor cells and form a structural meshwork in which photoreceptors are embedded [30]. The stalk of the Müller cell cone does not contribute to the formation of the ELM because the cells of the Müller cell cone have no direct contact to photoreceptor cells [1, 5]. This ensures that the ELM remains nondisrupted after a disruption of the stalk of the Müller cell cone.

A schistic splitting of the foveal walls between the OPL and HFL in tractional lamellar holes, foveal pseudocysts, outer lamellar holes, and macular pseudocysts is produced by anteroposterior (Figs. 1a, e, f and 2b‒h) and/or tangential tractions (Figs. 1b, c and 2a, j, k). These tractions may cause a disruption of the horizontal layer of the Müller cell cone or of the connection between this layer and the foveal walls (Fig. 1a, e, f), resulting in an elevation of the inner layers of the foveal walls which produces the schistic tissue splitting (Fig. 8b, c). The schistic spaces are kept together by bundles of Henle fibers. The low mechanical stability of the OPL-HFL interface in the foveal walls and parafovea (Fig. 8a) may explain the locations of the tissue splitting between the OPL and HFL in tractional lamellar holes and of the cavitations into the lower foveal walls in degenerative lamellar holes (Fig. 8c, d). During the ontogenetic development of the fovea, the OPL-HFL interface is a flexible stratum which allows the counter-movement of the inner and outer retina by a passive tractional elongation of the Henle fibers [4]. This versatile sliding zone may also facilitate tissue movements implicated in the adaptation of the shape of the mature fovea to the lighting conditions. It was suggested that Müller cell-mediated morphological alterations of the foveal shape, resulting in flattening and deepening of the foveal pit, may contribute to the adaptation of the position of the central photoreceptors to changes in the angle of the incoming light, and thus may play a role in accomodation and fixation (Fortin, 1925, cited in Kolmer and Lauber [31]). These morphological alterations of the foveal shape were suggested to be mediated by tractional forces exerted by the Müller cells of the foveal walls onto the Henle fibers which result from a contraction or relaxation of the horizontal Müller cell side processes in the inner part of the OPL [1]. To allow such morphological alterations of the fovea, the Henle fibers are not connected; single or several Henle fibers can shift against the others [1]. The structural tissue stabilization is supported by the strands of microtubules and intermediate filaments like vimentin and glial fibrillary acidic protein (GFAP) in Müller cells [4, 32, 33]. It was shown in the fovea of macaques that the outer part of the OPL is largely devoid of vimentin and that the OPL-HFL interface is devoid of GFAP while the Müller cell processes in the inner part of the OPL, the HFL, and the ONL contain vimentin and GFAP [1, 4]. The absence of glial intermediate filaments at the OPL-HFL interface may explain the low mechanical resistance of this interface against tractional forces. A further possibility which may explain the low mechanical stability of the OPL-HFL interface is that tractional forces can easily disrupt the photoreceptor synapses in the outer part of the OPL and/or the connections between the synapses and the photoreceptor cell axons.

The present data may suggest that one function of the Müller cell cone is to provide the stability of the foveal structure by the prevention of an elevation of the inner layers of the foveal walls which may result in a schistic splitting of the tissue between the OPL and HFL in the foveal walls. We found in one eye a schistic splitting between the OPL and ONL in the peripapillary retina (Fig. 4f); this may suggest that a low mechanical stability of the interface between the OPL and HFL/ONL may be a phenomenon not restricted to the macular region.

### Pathogenesis of schistic and degenerative cavitations in lamellar holes

The contours of the schistic and degenerative cavitations in the foveal walls are different in tractional and degenerative lamellar holes. In tractional holes, the schistic cavities between the OPL and HFL are tapered and have a more or less regular contour (Fig. 8c). On the other hand, the cavitations into the lower foveal walls in degenerative holes have a more irregular morphology (Fig. 8d). The schisis between the OPL and HFL is likely caused by the tractional elevation of the inner layers of the foveal walls (NFL to OPL) (Fig. 8b, c).

It was suggested that tractional forces, exerted by contractile ERM and/or the partially detached posterior hyaloid, are the primary cause of tractional lamellar holes [14, 18, 20] whereas degenerative lamellar holes result from a slow and chronic degenerative process [9]. Normally, the Müller cell cone in the foveola prevents an elevation of the inner layers of the foveal walls while Müller cells of the foveal walls provide the stability of the outer layers of the fovea [1]. One pathogenic event implicated in the development of tractional and degenerative lamellar holes is a tractional disruption of the Müller cell cone or of the connection between the Müller cell cone and the foveal walls which allows an elevation of the inner layers of the foveal walls that is associated with the formation of a schisis between the OPL and HFL or of degenerative cavitations into the lower foveal walls. The traction may also cause the formation of a foveal pseudocyst characterized by a detachment of the inner Müller cell layer from the HFL/ONL in the foveola; because the horizontal layer of the Müller cell cone is laterally connected to the foveal walls, the elevation of the inner foveolar layer is associated with an elevation of the inner layers of the foveal walls (Fig. 8b). Disruption of the elevated Müller cell cone may produce a tractional lamellar hole from a foveal pseudocyst.

The pathogenesis of degenerative lamellar holes is largely unclear. We found that degenerative cavitations display the widest lateral extensions at two levels: the OPL-HFL interface and the IPL-INL interface (Fig. 7b), and that degenerative lamellar holes are characterized by degenerations of the HFL, OPL, and INL, which is reflected in the shape of the degenerative cavitations, and by a (partial) degeneration of the central ONL and photoreceptor layer (Fig. 8d). The two-level shape of the degenerative cavitations and the degenerations of different layers may suggest that various pathogenic processes contribute to the development of the cavitations (Fig. 8h). We propose that the development of the cavitations in degenerative lamellar holes is initiated by traction which disrupts the Müller cell cone or the connection between the Müller cell cone and the foveal walls, resulting in an indentation of the foveal pit between the OPL and HFL; the indentation subsequently enlarges to a small schisis between the OPL and HFL, as shown in the example of Fig. 3e. The schisis causes a slow and chronic degeneration of Henle fibers or of the connection between the photoreceptor synapses in the OPL and the photoreceptor axons in the HFL. This results in a degeneration of the photoreceptor synapses in the outer part of the OPL. The degeneration of the photoreceptor synapses induces a retrograde degeneration of horizontal cells in the inner part of the OPL and bipolar cells in the INL, resulting in a degeneration of the OPL and INL. The degeneration of the OPL and INL explains the presence of a second level of the lateral extension of the cavitations at the IPL-INL interface (Fig. 7b). The outer layer of the elevated foveal walls above the degenerative cavitations is the IPL because retinal ganglion cells, which have their dendritic trees in this layer, are not affected. The absence of a degeneration of retinal ganglion cells is also suggested by the permanent presence of the NFL which contains the axons of the cells, and might be explained with the fact that the survival of retinal ganglion cells also depends on trophic factors supplied by their central target structures, in addition to the local trophic support [34–36]. The disruption of Henle fibers also results in a degeneration of photoreceptor cells resulting in the defects of the central ONL and photoreceptor layer. This mechanism may explain the correlation between the horizontal diameter of the degenerative cavitations and the defect of the photoreceptor layer [37]. In addition, the degeneration of central photoreceptor cells may induce a proliferation of the cells of the disrupted Müller cell cone in the foveola which fill the spaces left by degenerated photoreceptors. As shown in the example of Fig. 3e, the degeneration of central photoreceptor cells (indicated by the thinning of the central ONL; 23 months) precedes the development of the Müller cell tissue in the foveola.

There is no or less degeneration of Henle fibers in tractional lamellar holes; bundles of nondisrupted Henle fibers traverse the schistic cavities between the OPL and HFL (Figs. 1a‒e and 7a). Because there is no degeneration of Henle fibers in tractional lamellar holes, there are no degenerations of the HFL, OPL, and INL, and no defects of the central ONL and photoreceptor layer, and the outer layer of the elevated foveal walls above the schistic spaces is the OPL (Fig. 8c). It was shown that the maximal visual acuity depends upon the density of the central photoreceptors [38]. Because the central photoreceptors and the Henle fibers are not degenerated in tractional lamellar holes, the visual acuity of eyes with tractional lamellar holes is higher than that of eyes with degenerative lamellar holes [8, 16, 20, 39, 40]. The proposed model suggests that both tractional and degenerative processes contribute to the development of degenerative lamellar holes while only traction produces the formation of tractional lamellar holes.

In degenerative lamellar holes of several patients, the elevated inner layers of the foveal walls protruded nearly straightly above the foveal pit, and the base of the protruded walls was formed by the IPL (Figs. 2a‒c, e, f, 5, and 7b). Because the IPL contains a dense network of horizontal neuronal and glial interconnections, it has a higher stiffness than the ganglion cell layer and INL [1] and thus may provide the stability of the protruded walls.

It is unclear why Henle fibers degenerate in degenerative but not in tractional lamellar holes. Henle fibers are composed of photoreceptor axons which are surrounded by the outer processes of the Müller cells of the foveal walls [4, 5]. Although the central ELM, which is formed by junctions between photoreceptor cells and the outer processes of the Müller cells of the foveal walls [28], shows morphological alterations due to the degeneration of photoreceptor cells and the proliferation of the cells of the Müller cell cone, the central ELM is not disrupted in most cases of degenerative lamellar holes. The integrity of the ELM may suggest that the degeneration of the Henle fibers is caused by a disruption of the photoreceptor axons but not of the outer processes of the Müller cells of the foveal walls. It could be that variations of structural proteins in the photoreceptor axons like neuronal filaments may determine whether tractional forces result in the development of a tractional or degenerative lamellar hole.

### LHEP and Müller cells

LHEP on the vitreal surface of the foveal walls and parafovea were found in eyes with degenerative macular holes (Figs. 3a‒f, 4a‒e, 5, and 7b). All cases of tractional lamellar holes (Figs. 1a‒f and 7a), foveal pseudocysts (Fig. 2a‒e), outer lamellar holes (Fig. 2f‒h), and macular pseudoholes (Fig. 2i‒k) investigated in this study were without LHEP. These data are in agreement with previous studies which showed that LHEP is most frequently observed in eyes with degenerative lamellar holes; some cases of other types of partial-thickness macular defects and FTMH (Figs. 3f and 6) may also exhibit LHEP [9, 15, 16, 20, 21, 41]. The time-dependent enlargement of LHEP was shown to be associated with an enlargement of the degenerative cavitations into the lower foveal walls [37, 39]. This association suggests that the degeneration of the Henle fibers could also represent a trigger which induces the development of LHEP. Because there is no degeneration of Henle fibers in tractional lamellar holes, LHEP does normally not develop in this type of lamellar holes [9, 10].

LHEP is composed of a hyperreflective inner layer and a tissue of homogenous medium reflectivity between this layer and the hyperreflective NFL. The functional role and pathogenesis of LHEP are unclear. The main cellular constituents of LHEP were suggested to be glial cells and vitreal cells like fibroblasts and hyalocytes [14, 16, 17, 42]. The origin of the macular pigment-containing glial cells in LHEP [14, 15, 17] is unclear. These cells may represent Müller cells of the foveal walls which were disrupted within the HFL and migrated onto the retinal surface and proliferated here [15, 17, 19]. Proliferating astrocytes may contribute to LHEP formation in the parafovea. Because the Müller cell cone contains the highest density of macular pigment [43], it is likely that glial cells in LHEP are also derived from the Müller cell cone. It was described that Müller cells in the foveola show a morphological connection with LHEP [11, 16, 21]. In the present study, we show cases of degenerative lamellar holes and FTMH which displayed morphological continuities between Müller cells in the foveola and the middle part of LHEP (Figs. 4a–f, 5, and 6). The data suggest that one component which forms LHEP are hypertrophied and/or proliferating and migrating cells of the Müller cell cone (Fig. 8d). The finding that connections between the foveolar Müller cells and LHEP are mainly present in nonelevated foveal walls (Figs. 4a‒f and 5) may suggest that these connections stabilize the foveal structure by the prevention of an elevation of the inner layers of the foveal walls which otherwise may cause an enlargement of the degenerative cavitations. In the FTMH shown in Fig. 6, Müller cells of the foveola and LHEP created a central plug which prevented the elevation of the foveal walls and thus the enlargement of the hole.

In all cases described in this study, Müller cells in the foveola were connected to the middle part of LHEP. Both displayed a medium reflectivity. This may suggest that Müller cells of the foveola contribute to the formation of this part of LHEP. As indicated by the morphological connection between vitreous remnants and LHEP in the foveal tissue of the case shown in Fig. 4f, a further cellular component which contributes to the development of the middle part of LHEP represents vitreal cells [14, 16, 17, 42]. The disruptions of the macular pigment-containing Henle fibers and Müller cell cone may explain the higher blue-fundus autofluorescence levels found in the foveal center of degenerative compared to tractional lamellar holes [40].

### LHEP and ERM

As previously shown [19, 41], tractional ERM and LHEP (Fig. 4e) and/or vitreomacular adhesion and LHEP (Figs. 3a and 4a, f) may coexist in degenerative lamellar holes. Whether this coexistence reflects a causal relationship is unclear. Pang et al. [16] showed in one eye with a developing degenerative lamellar hole that a tractional ERM in the parafovea was replaced by LHEP. Morphological continuities between ERM or the partially detached posterior hyaloid and the inner hyperreflective layer of LHEP were previously described in eyes with degenerative lamellar holes [11, 12, 16, 19, 20]. A continuity between the hyperreflective innermost layer in the parafovea, which laid above the NFL, and the inner hyperreflective layer of LHEP was also observed in many cases investigated in this study. ERM may continue into the inner or both hyperreflective layers of LHEP (Figs. 4f and 6). These continuities may suggest that the vitreal hyperreflective layer of LHEP is constituted by a transformed ERM and/or the attached posterior hyaloid. The outer hyperreflective layer of LHEP may be also formed by a gliotic NFL (Fig. 3a); the gliosis may involve both retinal astrocytes and Müller cell endfeet. As previously proposed [44], LHEP may develop in response to tractions exerted by ERM or the partially detached posterior hyaloid to relieve the tractional forces and to stabilize the shape of the foveal walls. However, there are no LHEP in most cases of tractional lamellar holes which can be also produced by tangential traction exerted by ERM. Perhaps, this can be explained with the absence of a degeneration of the central outer retina and Henle fibers. Further research is required to determine the various factors which contribute to the pathogenesis of the various types of partial-thickness macular defects.

## Conclusions

Tractional and degenerative lamellar holes are produced by traction exerted by the partially detached posterior hyaloid and/or contractile ERM. One of the first steps of hole formation is likely a disruption of the Müller cell cone in the foveola or of the connection between the cone and the foveal walls. However, the different morphologies of the schistic and degenerative cavitations in tractional and degenerative lamellar holes, in association with (degenerative holes) or not (tractional holes) a degeneration of central photoreceptor cells, may suggest a partly different pathogenesis of both types of lamellar holes. The schistic splitting of the foveal walls in tractional lamellar holes is likely produced by traction which causes an elevation of the inner layers of the foveal walls. The location of the schisis can be explained with sites of tissue layer interfaces with low mechanical stability: the boundary with no cellular connections between both Müller cell populations in the foveola, and the interface between the OPL and HFL in the foveal walls and parafovea. A similar mechanism may also explain the formation of the schistic cavities in foveal pseudoholes, outer lamellar holes, and some cases of macular pseudoholes. We propose that the development of the cavitations in degenerative lamellar holes is initiated by traction which produces a schisis between the OPL and HFL, and enlarged by a slow and chronic degeneration of Henle fibers; this may cause a subsequent degeneration of the central photoreceptor cells and a retrograde degeneration of horizontal and bipolar cells. However, further research is required to reveal the etiologies and pathogenic steps implicated in the development of the different types of partial-thickness macular defects.

## Data Availability

All data generated or analysed during this study are included in this published article.

## Abbreviations

BCVA: Best-corrected visual acuity
ELM: External limiting membrane
ERM: Epiretinal membrane
EZ: Ellipsoid zone
FTMH: Full-thickness macular hole
GFAP: Glial fibrillary acidic protein
HFL: Henle fiber layer
INL: Inner nuclear layer
IPL: Inner plexiform layer
IZ: Interdigitation zone
LHEP: Lamellar macular hole-associated epiretinal proliferation
NFL: Nerve fiber layer
ONL: Outer nuclear layer
OPL: Outer plexiform layer
RPE: Retinal pigment epithelium
SD-OCT: Spectral-domain optical coherence tomography
